# Paper Synthesis, Cytotoxicity and Apoptosis Induction in Human Tumor Cells by Galaxamide and Its Analogues

**DOI:** 10.3390/md12084521

**Published:** 2014-08-18

**Authors:** Xi Xiao, Xiaojian Liao, Shaoling Qiu, Zihao Liu, Bin Du, Shihai Xu

**Affiliations:** 1 Department of Chemistry, Life Science School, Jinan University, Guangzhou 510632, China; E-Mails: xxiaoxi0312@163.com (X.X.); tliaoxj@jnu.edu.cn (X.L.); qslhappyrain@gmail.com (S.Q.); 2 Department of Pathology, Medical School, Jinan University, Guangzhou 510632, China; E-Mail: mercuryliu@sina.cn

**Keywords:** galaxamide, peptide, synthesis, anticancer, apoptosis

## Abstract

Our previous study reported that galaxamide, which is a cyclo-pentapeptide containing five leucines that was extracted from *Galaxaura filamentosa*, displayed remarkable anticancer cytotoxicity. This novel cyclo-peptide provided a new skeleton for the structural modifications used in finding new drugs with better anticancer properties. In this study, five analogues were synthesized based on changing the number of d/l amino acids by adding a new amino acid, phenylalanine. Galaxamide and five of its analogues were evaluated through MTT assays to examine their cytotoxic activities. We found that modified analogue 5, which is referred to as **A5**, displayed broad spectrum cytotoxic activity toward every cell line tested; in addition, the IC_50_ of **A5** was lower than that of galaxamide and the other analogues. Furthermore, we used flow cytometry and western blot assays to investigate whether galaxamide and **A5** could induce cancer cell apoptosis. The flow cytometric studies showed that HepG2 cells treated with different concentrations of galaxamide or **A5** over 72 h displayed significant and dose-dependent increases in the percentages of early-stage apoptotic cells. Western blotting revealed that both compounds induce caspase-dependent apoptosis in HepG2 cells through a mitochondria-mediated pathway. The results demonstrate that galaxamide and its analogues have potential applications as clinical anticancer drugs.

## 1. Introduction

**Figure 1:**
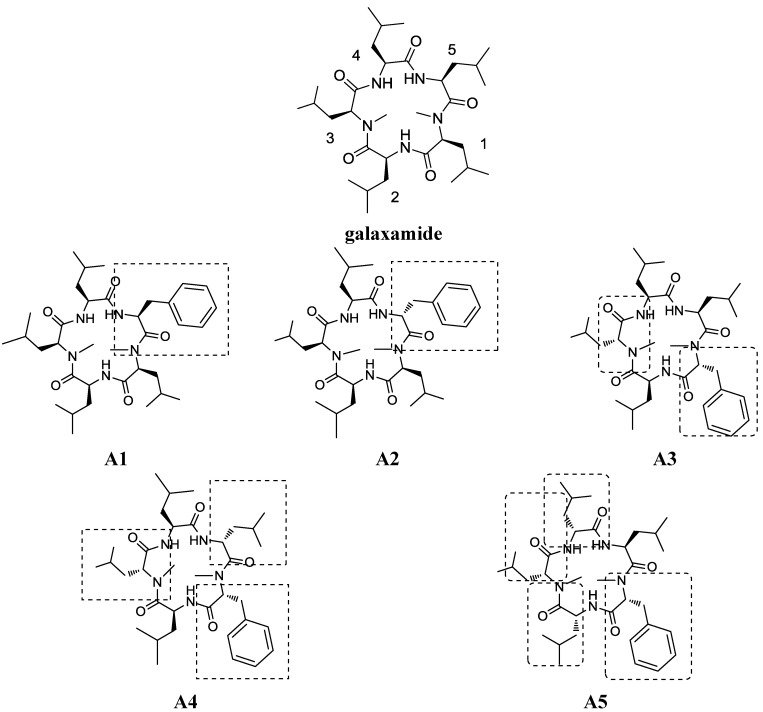
Structure of galaxamide and galaxamides **A1**–**A5**.

The ocean has more species diversity than land, supplying numerous organisms with chemical properties that are different from their terrestrial counterparts [1]. Cyclic peptides are an important class of marine compounds that may exhibit strong antitumor, antiviral and antibiotic activities [2]. Many structurally novel bioactive cyclopeptides have been isolated from marine cyanobacteria in recent decades [3]. Cyclic peptides tend to have a greater binding affinity for protein targets than their linear counterparts or small molecules, because their bond rotation is restricted and their structures are conformationally constrained [4]. Galaxamide is a novel cyclic pentapeptide isolated from *Galaxaura filamentosa* in the South China Sea, Xisha Islands. Our earlier research resulted in the isolation, structural determination and total synthesis of galaxamide, revealing significant antitumor activity against the human renal cell carcinoma GRC-1 and hepatocellular carcinoma HepG2 cell lines [5]. The pharmacological and toxicological profiles of galaxamide strongly suggested that galaxamide might be a promising anticancer drug candidate. Therefore, we modified galaxamide and investigated its structure-activity relationship to discover novel antitumor agents among the galaxamide analogues. In this work, we changed the number of d-amino acids in galaxamide [6] and different residues, including leucine and phenylalanine, which is considered an active group in cyclopentapeptide [7], were replaced to synthesize analogues 1–5 (referred to as **A1**–**A5**, Figure 1). All of the compounds were evaluated for their cytotoxicity against four human cancer cell lines, HepG2, MCF-7, SW480 and U87, as well as normal human liver cells (L02). As expected, several modified compounds showed strong antitumor activities; in particular, **A5** demonstrated stronger cytotoxic activities than galaxamide against all of the tested cancer cell lines. Furthermore, we demonstrated that galaxamide and **A5** could activate caspase-3, caspase-9 and PARP, inducing apoptosis in HepG2 and elucidating the mechanism of its anti-tumor activity.

## 2. Results

### 2.1. Chemistry

**Scheme 1:**
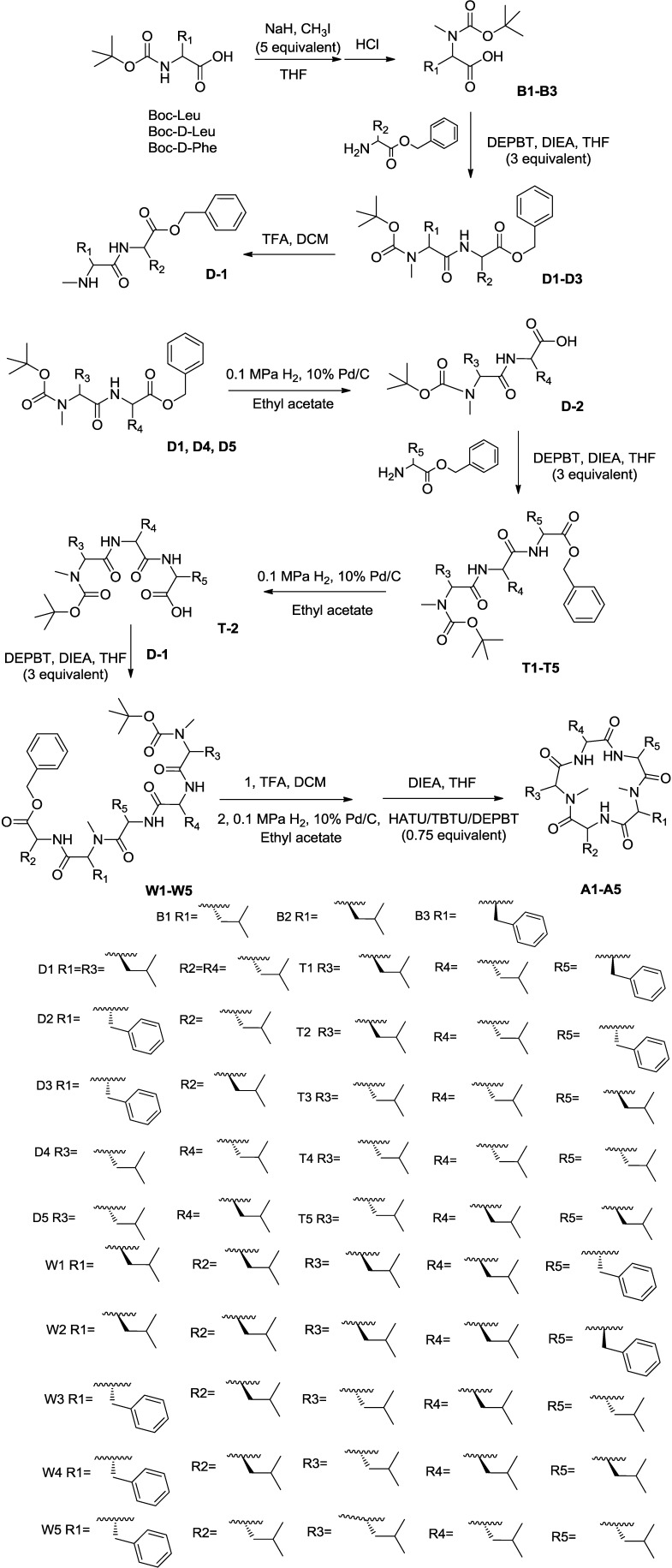
The synthesis of galaxamides **A1**–**A5**. DEPBT, 3-(diethoxyphosphoryloxy)-1,2,3-benzotriazin-4(3H)-one; DIEA, diisopropylethylamine; DCM, dichloromethane.

In our previous paper, we described the synthesis of galaxamide, which is a cyclic peptide [5]. We employed the same solution phase method to synthesize galaxamides **A1**–**A5**, as shown here (Scheme 1) for **A5**.

Following the strategy as Sakurai [8] described, our synthesis began with the commercially available *N*-(tert-butoxycarbonyl)-leucine, *N*-(tert-butoxycarbonyl)-d-leucine and *N*-(tert-butoxycarbonyl)-d-phenylalanine, reacting with CH_3_I/NaH [9] in THF, generating Compounds **B1**–**B3**, respectively. A reaction between **B1**–**B3** and amino acids protected by benzyl ester in the presence of a coupling reagent, specifically 3-(diethoxyphosphoryloxy)-1,2,3-benzotriazin-4(3*H*)-one (DEPBT) and diisopropylethylamine (DIEA), generated dipeptides **D1**–**D5** in an 85.0%–92.0% yield [10]. Additionally, we removed the t-butyl carbamate (referred to as Boc) group from **D1**–**D3** using TFA to produce **D-1**. A hydrogen reduction over Pd/C [11] removed the benzyl group from **D1**, **D4** and **D5** before amino acids protected by benzyl ester were coupled using DEPBT, generating tripeptides **T1**–**T5** in an 83%–88% yield. Subsequently, the benzyl group was removed to obtain **T-2**. **C-1** coupled with **T-2** via DEPBT, generating pentapeptides **W1**–**W5**. Each coupling reaction was concentrated upon completion and subjected directly to silica gel column chromatography using *n*-hexane/acetone (20:1).

The cyclization is a key step during the synthesis of cyclic peptides. After removing the Boc group and benzyl groups from pentapeptides **W1**–**W5** using TFA and a hydrogen reduction over Pd/C, respectively, the dried, unpurified free amine/free acid linear pentapeptide was dissolved in 2:2:1 THF/CH_3_CN/CH_2_Cl_2_ (0.003 M). Afterwards, three coupling agents, specifically DEPBT, 2-(1*H*-7-azabenzotriazol-1-yl)-1,1,3,3-tetramethyluronium hexafluorophosphate (HATU) and *O*-(benzotriazol-1-yl)-*N*,*N*,*N*′,*N*′-tetramethyluronium tetrafluoroborate (TBTU) (0.75 equivalent each) with DIEA (six equivalent) were added to the reaction [12,13]. The reaction was usually completed within approximately four days. The final product was generated after removing the solvent by distillation under reduced pressure. The mixture was dissolved in EtOAc and washed with saturated ammonium chloride solution [5]. The organic layers were dried, filtered and concentrated. The unpurified mixture was subjected to reverse phase HPLC to obtain **A1**–**A5** in a 44.0%–47.0% yield.

### 2.2. Biological Activity

#### 2.2.1. Galaxamide and Its Analogues Inhibit the Proliferation of Different Cancer Cells

To examine the effect of galaxamide and its five analogues on cell proliferation, four cancer cell lines and one normal cell line, including human hepatocellular carcinoma HepG2, human breast carcinoma MCF-7, human colon carcinoma SW480 and human glioma cell line U87, as well as human normal liver cell line L02, were exposed to all of the compounds at different concentrations before being subjected to an MTT assay. Galaxamide and **A1**–**A5** were cytotoxic toward different cancer cell lines while exhibiting low cytotoxicity toward normal liver cell line L02. All of the tested compounds exhibited strong cytotoxic activity against HepG2 cells. When comparing galaxamide and its five analogues, **A5** demonstrated the highest cytotoxicity against every cell line. The results are summarized in Table 1 and discussed below.

**Table 1:** Cytotoxicity of compounds galaxamide and **A1**–**A5** against HepG2, L02, SW480, U87 and MCF-7 cells (IC_50_, μg/mL) ^a^.

Compound/Cell Line	HepG2 ^b^	L02 ^b^	SW480 ^b^	U87 ^b^	MCF-7 ^b^
Galaxamide	4.63 ± 0.14	>40	>40	10.61 ± 0.24	14.09 ± 1.35
**A1**	4.11 ± 0.16	>40	12.37 ± 1.01	>40	9.40 ± 0.43
**A2**	4.56 ± 0.20	>40	5.75 ± 0.19	>40	9.64 ± 0.35
**A3**	5.58 ± 0.11	>40	10.20 ± 0.15	20.16 ± 0.30	7.23 ± 0.27
**A4**	6.27 ± 0.13	>40	8.60 ± 0.08	2.82 ± 0.10	6.56 ± 0.18
**A5**	1.46 ± 0.09	10.55 ± 0.60	5.10 ± 0.12	1.85 ± 0.11	4.18 ± 0.15

^a^ The IC_50_ values are reported as the means ± the standard error from three independent experiments; ^b^ HepG2, human hepatoma cancer cell line; L02, human normal liver cell line; SW480, human colon carcinoma cell line; U87, human Glioma cell line; MCF-7, human breast cancer cell line.

#### 2.2.2. Effect of Galaxamide and **A5** on the Cell Morphology and Nuclear Integrity

**Figure 2:**
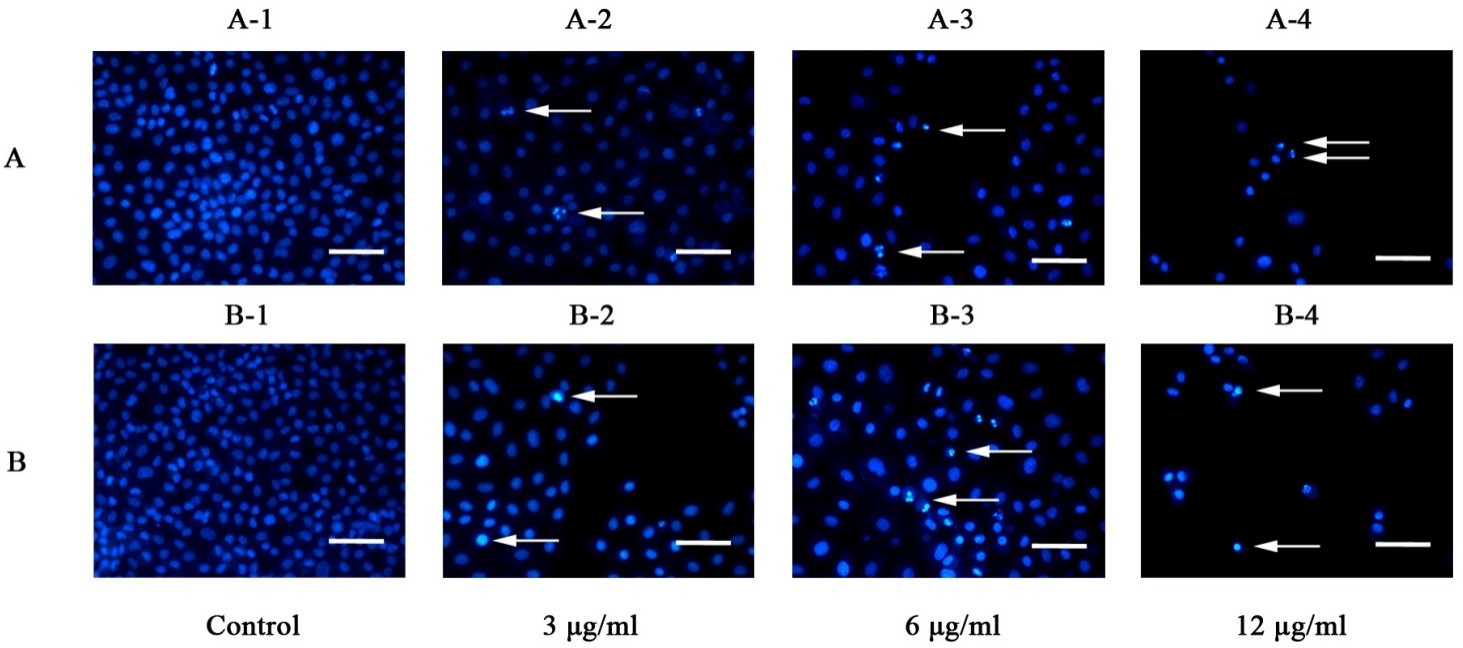
Morphology observation of galaxamide and **A5**-induced apoptosis, respectively, in HepG2 cells as indicated by Hoechst33342 staining. Cells were incubated in the absence or presence of 3, 6 and 12 μg/mL galaxamide (**A**) and **A5** (**B**). HepG2 cells were fixed and stained with Hoechst33342 to visualize the nucleus (blue) under a fluorescent microscope at a magnification of 200×; scale bar, 50 μm. Arrows indicate apoptotic features (condensed chromatin and nuclear fragmentation). Results are representative of triplicate experiments.

Galaxamide and **A5** induced morphological changes in HepG2 cells. Treating HepG2 cells with 3, 6, 12 μg/mL of galaxamide and **A5** for 72 h resulted in the detachment of most cells from the tissue culture plates and cell death. These behaviors might be caused by cytotoxicity toward the cells. The number of cells detached from the culture plates increased when increasing the concentrations of galaxamide and **A5** in the plates. To examine whether the cells died through apoptotic mechanisms, the control and the galaxamide- and **A5**-treated cells were stained with Hoechst33342. The resulting morphological changes were observed under a fluorescence microscope (Figure 2). The control cells exhibited normal morphologies with round nuclei. However, the galaxamide- and **A5**-treated cells showed chromatin condensation at the edges of the nuclear membrane and nuclear fragmentation. As the concentration of galaxamide and **A5** was increased to 12 μg/mL, most of them were stimulated to death, but morphological changes still could be observed.

#### 2.2.3. Galaxamide and **A5** Induce HepG2 Apoptosis

**Figure 3:**
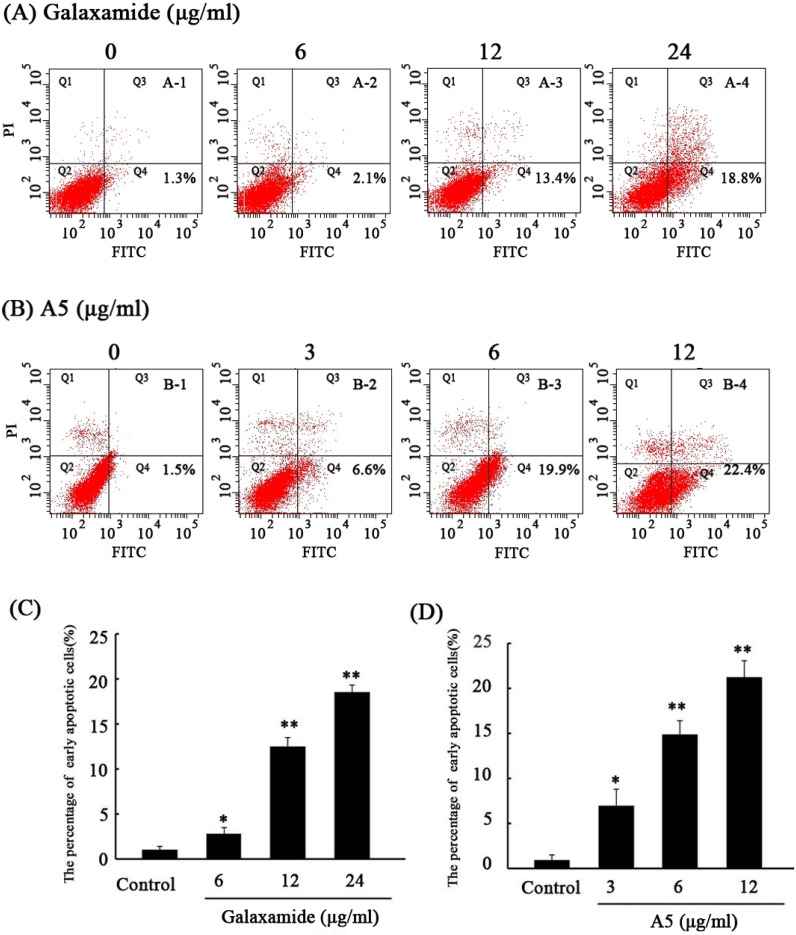
Galaxamide and **A5** affect the apoptosis of HepG2. HepG2 cells were treated with galaxamide or **A5** at different concentrations for 72 h. The control and treated HepG2 cells were assessed using Annexin V-fluorescein isothiocyanate (FITC) and propidium iodide (PI) dual parameter flow cytometry. A dual parameter dot plot of FITC-fluorescence (*x*-axis) *vs.* PI-fluorescence (*y*-axis) shows a logarithmic intensity. A dual parameter dot plot of FITC-fluorescence (*x*-axis) *vs.* PI-fluorescence (*y*-axis) shows a logarithmic intensity. Quadrants: lower left, the live cells; lower right, the early apoptotic cells; upper left, the necrotic cells; and upper right, late apoptotic cells. (**A**,**B**) Representative Annexin V/PI double staining in the galaxamide (**A**) or **A5** treatment group (**B**); and (**C**,**D**) a comparison of the percentages of early apoptosis in the galaxamide (**C**) or **A5** treatment group (**D**). * *p* < 0.05 or ** *p* < 0.01 *vs.* the control groups.

To study the mechanism of the anti-tumor effect, HepG2 was stained with Annexin V/PI and subjected to flow cytometry after treatment with galaxamide and A5 (MW = 627.5 g/mol) at 6, 12 and 24 μg/mL for 72 h. As shown in Figure 3, the lower right quadrants represent the early apoptotic cells. After 72 h of treatment with galaxamide (MW = 593.4 g/mol) at different concentrations (6, 12 and 24 μg/mL), the percentage of early apoptotic cells increased from 1.06% ± 1.0% to 2.3% ± 0.3%, 4.3% ± 1.1% and 18.8% ± 1.9%, respectively (*p* < 0.05 Figure 3A,C). Lower concentrations of A5 (3, 6 and 12 μg/mL) increased the percentage of early apoptotic cells from 1.5% ± 0.4% to 6.6% ± 1.7%, 16.6% ± 1.5% and 22.4% ± 2.4%, respectively (*p* < 0.05, Figure 3B,D). In addition, galaxamide and A5 induced early apoptosis in HepG2 dose-dependently. Therefore, galaxamide and A5 induced apoptosis in HepG2 cells.

#### 2.2.4. Galaxamide and **A5** Activate Caspase-3, Caspase-9 and PARP in HepG2

**Figure 4:**
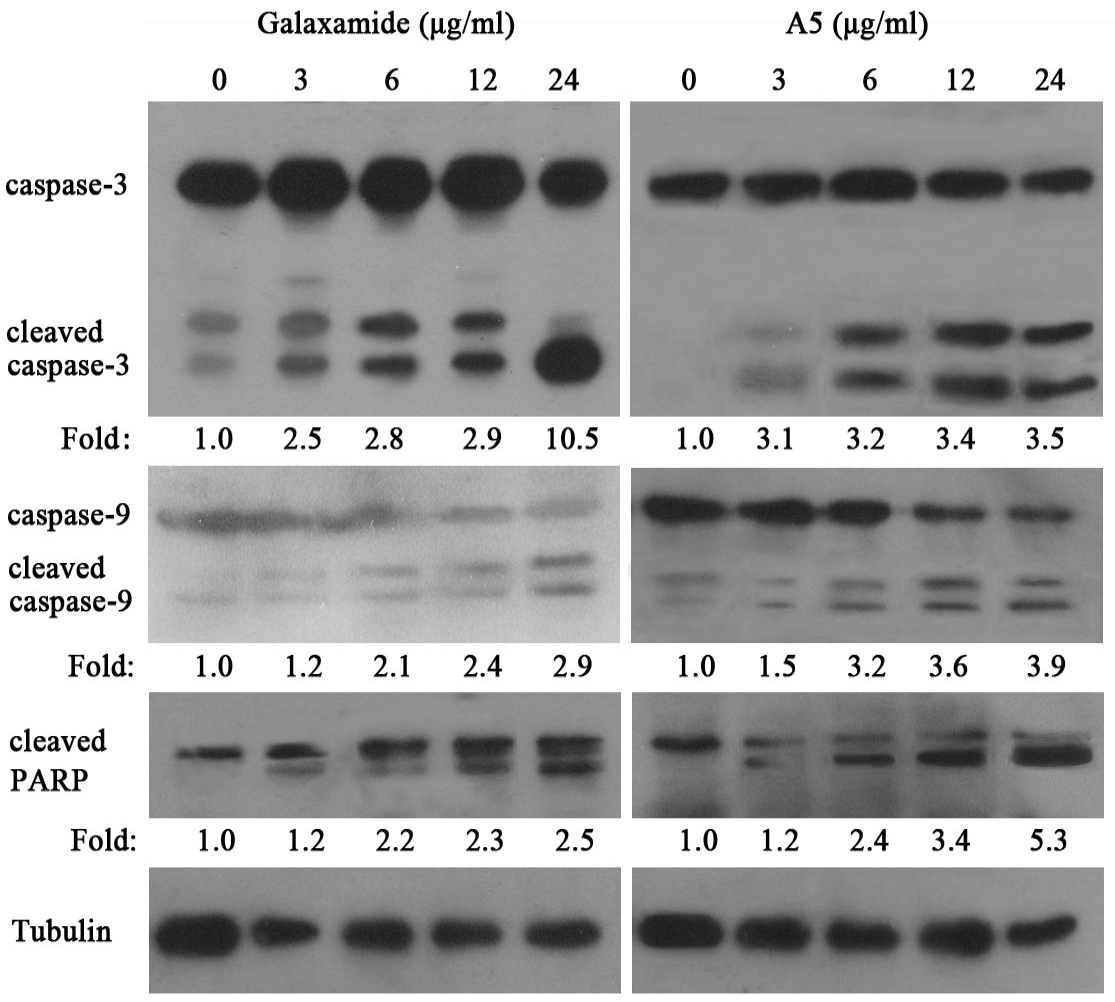
The expression of apoptosis-associated proteins in HepG2 cell treated with galaxamide and **A5** for 72 h. A western blot was used to analyze whole cell lysates for caspase-3, caspase-9 and cleaved PARP expression after treatment with galaxamide and **A5**, and equal amounts of the protein were loaded onto each lane. One representative immunoblot from three independent experiments is presented. The fold change was calculated as the ratio of cleaved caspase-3, cleaved caspasse-9 and cleaved PARP in the different concentration of Galaxamide or **A5** to their expression in the control cells, after normalization to tubulin in each lane. All data shown are representative of at least three separate studies.

Based on the increased apoptosis of galaxamide- and **A5-**treated cells, we next examined whether caspase-3, caspase-9 and PARP activities were changed after treatment with galaxamide and **A5**. As shown in Figure 4, the cleaved caspase-3, caspase-9 and PARP expressions were significantly increased after their treatment (3, 6, 12, and 24 μg/mL) for 72 h. These findings suggest that galaxamide and **A5** might induce apoptosis by regulating mitochondria-mediated pathways.

## 3. Discussion

In our previous paper [5], we described the isolation, structural determination and synthesis of galaxamide. In the present study, galaxamide and analogues **A1**–**A5** were synthesized; they all exhibited significant anti-tumor activity against different cancer cells. Furthermore, our data demonstrated that galaxamide and the best cytotoxic analogue, **A5**, could induce cell apoptosis through a mitochondria-mediated apoptosis pathway. Galaxamide and its analogues might be anti-tumor therapeutic agents ready for further study.

Galaxamide and **A1**–**A5** were tested for inhibitory activity against the growth of several tumor cell lines and one normal liver cell line. As shown in Table 1, some modified compounds exhibited much better activity than galaxamide. Notably, **A5** showed broad-spectrum antitumor activity against HepG2, SW480, U87 against MCF-7 cell lines with IC_50_ values of 1.46, 5.1, 1.85 and 4.18 μg/mL, which were 3.17-, 8.59-, 5.73- and 3.37-fold more active than galaxamide (with IC_50_ value of 4.63, 43.82, 10.61 and 14.09 μg/mL), respectively. Galaxamide and **A1**–**A4** exhibited low cytotoxic activity toward normal liver cell line L02 (IC_50_ > 40 μg/mL). Although **A5** achieved an IC_50_ value of 10.55 μg/mL, this value was about tenfold higher than that of HepG2. Galaxamide and its analogues affect cancer cells differently than normal cells. Moreover, all of the cyclopeptide exhibited strong antiproliferative activity against HepG2 cell, revealing that galaxamide and its analogues might be more effective against HepG2 than other types of tumor cells. Both **A1** and **A2** displayed better cytotoxic activities against HepG2, MCF-7 and SW480. **A3** and **A4** did not exhibited equal cytotoxic activity against HepG2 compared to galaxamide, but they performed better than galaxamide against SW480 and MCF-7; in addition, **A4** had an IC_50_ value of 2.82 μg/mL and was more active than galaxamide.

Relative to the structure, **A5** has four d-amino acids in Positions 2–5. In comparison, **A1** has phenylalanine, while **A2** is changed into a d-amino acid at Position 1. **A3** has two d-amino acids in Positions 2 and 4, and **A4** has three d-amino acids in Positions 1, 2 and 4. The structural analysis revealed that: (1) **A1** and **A2**, which contain d/l-phenylalanine, exhibited favorable activity; (2) **A5** was more potent than the other analogues, suggesting that the compounds with four d-amino acids, including a d-*N*-Me-phenylalanine, exhibit better activity than analogues bearing fewer d-amino acids than **A2**–**A4**. Future analogues that incorporate additional d-amino acids at the other positions will be synthesized and tested.

Apoptosis is an important homeostatic mechanism that balances cell division and death while maintaining the appropriate number of cells in the body [14]. Many therapeutic peptides, such as aplidine and didemnin B, which both were isolated from marine animals, could induce apoptosis in cancer cells [15,16]. Cyclopentapeptide sansalvamide A is a derivative of sansalvamide A, which is a depsipeptide with antitumor activity that promotes apoptosis in cancer cells [17]. Galaxamide and its analogues were also peptide compounds. In the present study, Annexin V/PI double-staining assays revealed that galaxamides and **A5** induced apoptosis in HepG2 cells. Therefore, inducing apoptosis might be the primary mechanism for the anti-cancer activity of galaxamides. In addition, a lower concentration of **A5** induced a better apoptosis effect than galaxamides. Similar to the results of the cytotoxicity experiments, we believe that **A5** has stronger anti-cancer activity.

Apoptosis can be induced through two distinct pathways [18]: the ligation of the TNF/Fas-receptor with its ligand followed by caspase-8 activation to activate caspase-3 directly and the mitochondria-mediated caspase-9 activation pathway. Initiator caspases cleave procaspases are able to activate effector caspases (caspase-3, -6 and -7) or amplify the caspase cascade by increasing the activation of the initiator caspases. Both pathways converge at caspase-3, leading to cell death. In the present study, caspase-3, caspase-9 and PARP were all activated in HepG2 after being treated with galaxamide or **A5**. Therefore, the apoptosis of HepG2 induced by galaxamide and **A5** might be related to a mitochondria-mediated pathway.

In summary, galaxamide and its analogues have potent antitumor effects and cause cell apoptosis *in vitro*. Future analogues that incorporate additional d-amino acids at other positions will be synthesized and tested. In addition, improving the yield of the cyclization is also necessary.

## 4. Experimental Section

### 4.1. Chemistry

The reagents and solvents were commercially available. The solvents were dried and purified through standard procedures before use. The NMR spectra were obtained on a Varian Inova-500 NB spectrometer in CDCl_3_, while using TMS as an internal standard; the coupling constants (*J*) are in Hz. The ESI mass spectra were obtained on a LCQ DECA XP LC-MS mass spectrometer. Column chromatography was performed using silica gel (Qingdao Ocean Chemical Factory, Qingdao, China, 200–300 mesh) eluted with petroleum ether/acetone or *n*-hexane/acetone; C18 reversed phase silica gel (Welch Material Inc., Shanghai, China, 45 μm) was eluted with methanol-water.

#### 4.1.1. *N*-Boc-Me-Leu-Leu-OBn (**D1**)

Yield: 90.0%; colorless crystal (CH_3_OH); ^1^H NMR (500 MHz, CDCl_3_): 7.36–7.40 (m, 5H), 6.51 (s, 1H), 5.16–5.20 (m, 2H), 4.65 (s, 2H), 2.75 (s, 3H), 1.63–1.68 (m, 4H), 1.50–1.54 (m, 2H), 1.48 (s, 9H), 0.90–0.96 (m, 12H); MS (ESI) *m*/*z*: 449.3 [M + H]^+^, 466.5 [M + NH_4_]^+^, 471.6 [M + Na]^+^.

#### 4.1.2. *N*-Boc-Me-d-Phe-Leu-OBn (**D2**)

Yield: 86.8%; colorless crystal (CH_3_OH); ^1^H-NMR (500 MHz, CDCl_3_), δ (ppm): 7.33–7.44 (m, 5H), 7.21–7.24 (m, 5H), 5.40 (d, *J* = 10.0 Hz, 1H), 5.22–5.25 (m, 1H), 5.10 (q, 2H), 4.92–4.97 (m, 1H), 2.97–3.01 (m, 2H), 2.70 (s, 3H), 1.63–1.69 (m, 1H), 1.54–1.58 (m, 1H), 1.48 (s, 9H), 0.93–0.96 (m, 1H), 0.84–0.90 (m, 6H); MS (ESI) *m*/*z*: 483.7 [M + H]^+^, 500.6 [M + NH_4_]^+^, 505.6 [M + Na]^+^.

#### 4.1.3. *N*-Boc-Me-d-Phe-d-Leu-OBn (**D3**)

Yield: 85.6%; colorless crystal (CH_3_OH); ^1^H-NMR (500 MHz, CDCl_3_), δ (ppm): 7.35–7.40 (m, 5H), 7.26–7.30 (m, 5H), 5.41 (d, *J* = 10.0 Hz, 1H), 5.24–5.30 (m, 1H), 5.11 (q, 2H), 4.94–4.97 (m, 1H), 3.01 (m, 2H), 2.70 (s, 3H), 1.64–1.69 (m, 1H), 1.57–1.60 (m, 1H), 1.48 (s, 9H), 0.93–0.96 (m, 1H), 0.84–0.88 (m, 6H); MS(ESI) *m*/*z*: 483.7 [M + H]^+^, 500.6 [M + NH_4_]^+^, 505.6 [M + Na]^+^.

#### 4.1.4. *N*-Boc-Me-d-Leu-Leu-OBn (**D4**)

Yield: 91.3%; colorless crystal (CH_3_OH); ^1^H-NMR (500 MHz, CDCl_3_), δ (ppm): 7.35–7.44 (m, 5H), 6.52 (s, 1H), 5.14–5.20 (m, 2H), 5.02 (d, 1H, *J* = 10.0 Hz), 4.61 (s, 2H), 3.04 (d, 2H, *J* = 5.0 Hz), 1.62–1.70 (m, 4H), 1.50–1.55 (m, 2H), 1.48 (s, 9H), 0.91–0.93 (m, 12H); MS (ESI) *m*/*z*: 449.3 [M + H]^+^, 466.5 [M + NH_4_]^+^, 471.6 [M + Na]^+^.

#### 4.1.5. *N*-Boc-Me-d-Leu-d-Leu-OBn (**D5**)

Yield: 92.0%; colorless crystal (CH_3_OH); ^1^H-NMR (500 MHz, CDCl_3_), δ (ppm): 7.37–7.47 (m, 5H), 6.50 (s, 1H), 5.14–5.22 (m, 2H), 5.02 (d, 1H, *J* = 10.0 Hz), 4.51 (s, 2H), 3.04 (d, 2H, *J* = 5.0 Hz), 1.66–1.72 (m, 4H), 1.49–1.55 (m, 2H), 1.38 (s, 9H), 0.91–0.93 (m, 12H); MS (ESI) *m*/*z*: 449.3 [M + H]^+^, 466.5 [M + NH_4_]^+^, 471.6 [M + Na]^+^.

#### 4.1.6. *N*-Boc-Me-Leu-Leu-Phe-OBn (**T1**)

Yield: 90.7%; colorless oil (CH_3_OH); ^1^H-NMR (500 MHz, CDCl_3_), δ (ppm): 7.32–7.37 (m, 5H), 7.22–7.26 (m, 5H), 7.14 (d, 2H, *J* = 6.8 Hz), 5.12 (s, 2H), 4.65–4.68 (m, 2H), 4.53– 4.56 (m, 1H), 2.98–3.01 (m, 2H), 2.42 (s, 3H), 1.59–1.63 (m, 2H), 1.47–1.53 (m, 2H), 1.43 (s, 9H), 1.38–1.42 (m, 2H), 0.86–0.91 (m,12H); MS (ESI) *m*/*z*: 595.7 [M + H]^+^, 612.7 [M + NH_4_]^+^, 617.9 [M + Na]^+^.

#### 4.1.7. *N*-Boc-Me-Leu-Leu-d-Phe-OBn (**T2**)

Yield: 91.5%; colorless crystal (CH_3_OH); ^1^H-NMR (500 MHz, CDCl_3_), δ (ppm): 7.32–7.40 (m, 5H), 7.24–7.30 (m, 5H), 6.78 (d, 1H, *J* = 10.0 Hz), 5.19 (d, 1H, *J* = 5.0 Hz), 4.73 (q, 1H), 4.49 (q, 1H), 2.95 (d, 2H, *J* = 10.0 Hz), 2.61 (s, 3H), 1.63–1.70 (m, 4H), 1.35 (s, 9H), 1.25–1.30 (m, 2H), 0.86–0.92 (m, 12H); MS (ESI) *m*/*z*: 595.7 [M + H]^+^, 612.7 [M + NH_4_]^+^, 617.9 [M + Na]^+^.

#### 4.1.8. *N*-Boc-Me-d-Leu-Leu-Leu-OBn (**T3**)

Yield: 87.1%; colorless oil (CH_3_OH); ^1^H-NMR (500 MHz, CDCl_3_), δ (ppm): 7.32–7.36 (m, 5H), 6.64 (d, *J* = 5.0 Hz, 1H), 6.56 (d, *J* = 5.0 Hz, 1H), 5.23 (d, *J* = 10.0 Hz, 1H), 5.16–5.19 (m, 2H), 4.69–4.73 (m, 1H), 4.53–4.56 (m, 1H), 2.97 (s, 3H), 1.87–1.93 (m, 2H), 1.53–1.56 (m, 4H), 1.42–1.46 (m, 9H), 1.36–1.39 (m, 3H), 0.92–0.96 (m, 18H); MS (ESI) *m*/*z*: 562.8 [M + H]^+^, 579.8 [M + NH_4_]^+^, 584.9 [M + Na]^+^.

#### 4.1.9. *N*-Boc-Me-d-Leu-Leu-d-Leu-OBn (T4)

Yield: 86.6%; colorless oil (CH_3_OH); ^1^H-NMR (500 MHz, CDCl_3_), δ (ppm): 7.37–7.47 (m, 5H), 6.65 (d, *J* = 5.0 Hz, 1H), 6.56 (d, *J* = 5.0 Hz, 1H), 5.25 (d, *J* = 10.0 Hz, 1H), 5.17–5.22 (m, 2H), 4.75–4.77 (m, 1H), 4.53–4.56 (m, 1H), 2.98 (s, 3H), 1.88–1.92 (m, 2H), 1.52–1.56 (m, 4H), 1.39–1.44 (m, 9H), 1.34–1.39 (m, 3H), 0.92–0.97 (m, 18H); MS (ESI) *m*/*z*: 562.8 [M + H]^+^, 579.8 [M + NH_4_]^+^, 584.9 [M + Na]^+^.

#### 4.1.10. *N*-Boc-Me-d-Leu-d-Leu-Leu-OBn (**T5**)

Yield: 87.2%; colorless oil (CH_3_OH); ^1^H-NMR (500 MHz, CDCl_3_), δ (ppm):7.35–7.40 (m, 5H), 6.60 (d, *J* = 5.0 Hz, 1H), 6.51 (d, *J* = 5.0 Hz, 1H), 5.19 (d, *J* = 10.0 Hz, 1H), 5.06–5.11 (m, 2H), 4.59–4.63 (m, 1H), 4.43–4.46 (m, 1H), 2.87 (s, 3H), 1.77–1.81(m, 2H), 1.43–1.52 (m, 4H), 1.32–1.37 (m, 9H), 1.24–1.27 (m, 3H), 0.88–0.96 (m, 18H); MS (ESI) *m*/*z*: 562.8 [M + H]^+^, 579.8 [M + NH_4_]^+^, 584.9 [M + Na]^+^.

#### 4.1.11. *N*-Boc-Me-Leu-Leu-Phe-Me-Leu-LeuOBn (**W1**)

Yield: 78.6%; colorless oil (CH_3_OH); ^1^H-NMR (500 MHz, CDCl_3_), δ (ppm): 7.33–7.41 (m, 7H), 7.20–7.24 (m, 3H) ,7.18 (d, 1H, *J* = 10.0 Hz), 6.68 (d, 1H, *J* = 10.0 Hz), 6.61 (d, 1H, *J* = 10.0 Hz), 5.24–5.30 (m, 2H), 5.13 (s, 2H), 5.06–5.11 (m, 1H), 4.93–4.99 (m, 2H), 2.91–2.95 (m, 2H), 2.83 (s, 3H), 2.73 (s, 3H), 1.77–1.80 (m, 2H), 1.65–1.68 (m, 6H), 1.53–1.59 (m, 4H), 1.45 (s, 9H), 0.93–0.99 (m, 14H), 0.84–0.88 (m, 10H); MS (ESI) *m/z*: 837.2 [M + H]^+^, 854.1 [M + NH_4_]^+^, 859.1 [M + Na]^+^.

#### 4.1.12. *N*-Boc-Me-Leu-Leu-d-Phe-Me-Leu-Leu-OBn (**W2**)

Yield: 81.0%; colorless oil CH_3_OH); ^1^H-NMR (500 MHz, CDCl_3_), δ (ppm): 7.31 (m, 7H), 7.23–7.26 (m, 3H) , 6.76 (d, 1H, *J* = 10.0 Hz), 6.36 (d, 1H, *J* = 10.0 Hz), 5.30 (d, 1H, *J* = 10.0 Hz), 5.13 (s, 2H), 5.01–5.05 (m, 1H), 4.83–4.90 (m, 2H), 4.70–4.73 (m, 1H), 4.56–4.59 (m, 1H), 2.96 (s, 3H), 2.73–2.77 (m, 2H), 2.67 (s, 3H), 1.64–1.67 (m, 2H), 1.59–1.62 (m, 6H), 1.46–1.50 (m, 4H), 1.41 (s, 9H), 0.88–098 (m, 24H), MS (ESI) *m/z*: 837.0 [M + H]^+^, 854.0 [M + NH_4_]^+^, 859.0 [M + Na]^+^.

#### 4.1.13. *N*-Boc-Me-d-Leu-Leu-Leu-d-Phe-Leu-OBn (**W3**)

Yield: 82.5%; colorless oil (CH_3_OH); ^1^H-NMR (500 MHz, CDCl_3_), δ (ppm): 7.35–7.45 (m, 7H), 7.26–7.30 (m, 3H), 6.76 (d, 1H, *J* = 10.0 Hz), 6.65 (d, 1H, *J* = 10.0 Hz), 6.37 (d, 1H, *J* = 10.0 Hz), 5.37 (s, 2H), 5.31–5.35 (m, 1H), 5.03–5.09 (m, 2H), 4.90–4.95 (m, 1H), 4.68–4.72 (m, 2H), 3.47 (s, 3H), 2.95 (s, 3H), 2.72–2.76 (m, 2H), 1.76–1.82 (m, 2H), 1.59–1.68 (m, 6H), 1.51–1.57 (m, 4H), 1.44 (s, 9H), 0.92–0.98 (m, 24H); MS (ESI) *m/z*: 837.0 [M + H]^+^, 854.0 [M + NH_4_]^+^, 859.0 [M + Na]^+^.

#### 4.1.14. *N*-Boc-Me-d-Leu-Leu-d-Leu-d-Phe-Leu-OBn (**W4**)

Yield: 81.5%; colorless oil (CH_3_OH); ^1^H-NMR (500 MHz, CDCl_3_), δ (ppm): 7.33–7.43 (m, 7H), 7.23–7.28 (m, 3H), 6.75 (d, 1H, *J* = 10.0 Hz), 6.64 (d, 1H, *J* = 10.0 Hz), 6.36 (d, 1H, *J* = 10.0 Hz), 5.35 (s, 2H), 5.31–5.33 (m, 1H), 5.03–5.10 (m, 2H), 4.88–4.92 (m, 1H), 4.68–4.75 (m, 2H), 3.57 (s, 3H), 2.97 (s, 3H), 2.74–2.78 (m, 2H), 1.76–1.81 (m, 2H), 1.57–1.66 (m, 6H), 1.53–1.55 (m, 4H), 1.45 (s, 9H), 0.91–0.95 (m, 24H); MS (ESI) *m/z*: 837.0 [M + H]^+^, 854.0 [M + NH_4_]^+^, 859.0 [M + Na]^+^.

#### 4.1.15. *N*-Boc-Me-d-Leu-d-Leu-Leu-d-Phe-d-Leu-OBn (**W5**)

Yield: 82.5%; colorless oil(CH_3_OH); ^1^H-NMR (500 MHz, CDCl_3_), δ (ppm): 7.35–7.45 (m, 7H), 7.26–7.30 (m, 3H), 6.76 (d, 1H, *J* = 10.0 Hz), 6.65 (d, 1H, *J* = 10.0 Hz), 6.37 (d, 1H, *J* = 10.0 Hz), 5.37 (s, 2H), 5.31–5.35 (m, 1H), 5.03–5.09 (m, 2H), 4.90–4.95 (m, 1H), 4.68–4.72 (m, 2H), 3.47 (s, 3H), 2.95 (s, 3H), 2.72–2.76 (m, 2H), 1.76–1.82 (m, 2H), 1.59–1.68 (m, 6H), 1.51–1.57 (m, 4H), 1.44 (s, 9H), 0.92–0.98 (m, 24H); MS (ESI) *m/z*: 837.0 [M + H]^+^, 854.0 [M + NH_4_]^+^, 859.0 [M + Na]^+^.

#### 4.1.16. Cyclo(Phe-*N*-Me-Leu-Leu-*N*-Me-Leu-Leu) (**A1**)

Yield: 42.0%; white powder (CH_3_OH); ^1^H-NMR (500 MHz, CDCl_3_), δ(ppm): 7.27–7.31 (m, 5H), 7.04 (d, *J* = 10.0 Hz, 1H), 6.93 (d, *J* = 10.0 Hz, 1H), 6.02 (d, *J* = 10.0 Hz, 1H), 5.17 (t, *J* = 15.0 Hz, 1H), 4.80–4.87 (m, 3H), 3.34 (dd, *J* = 5.0 Hz, 5.0 Hz, 1H), 3.20–3.27 (m, 6H), 2.65 (s, 3H), 1.94–2.00 (m, 1H), 1.66–1.75 (m, 2H), 1.47–1.59 (m, 7H), 1.17–1.22 (m, 1H), 0.90–0.98 (m, 24H); ^13^C-NMR (125 MHz, CDCl_3_), δ (ppm): 173.1, 172.3, 170.4, 170.2, 169.3, 137.4, 129.4(2C), 128.5(2C), 126.9, 64.2, 53.9, 53.2, 48.2, 47.8, 40.8, 40.6, 38.8, 36.9, 36.4, 34.6, 29.6, 25.2(2C), 24.7, 24.6, 23.3, 23.2, 22.8, 22.6(2C); MS (ESI) *m*/*z*/: 628.9 [M + H]^+^, 645.8 [M + NH_4_]^+^, 650.8 [M + Na]^+^.

#### 4.1.17. Cyclo(d-Phe-*N*-Me-Leu-Leu-*N*-Me-Leu-d-Leu) (**A2**)

Yield: 42.5%; white powder (CH_3_OH); ^1^H-NMR (500 MHz, CDCl_3_), δ(ppm): 8.13(s, 1H), 7.71–7.74 (m, 1H), 7.19–7.29 (m, 5H), 6.91 (d, *J* = 10.0Hz, 1H), 5.18 (t, *J* = 10.0Hz, 1H), 4.87–4.91 (m, 1H), 4.74–4.79 (m, 3H), 3.05–3.09 (m, 2H), 2.91–2.94 (m, 1H), 2.84 (s, 1H), 2.80 (s, 2H), 2.62 (s, 3H), 1.88–1.93 (m, 1H), 1.77–1.82 (m, 1H), 1.50–1.61 (m, 6H), 1.37–1.43 (m, 1H), 1.06–1.12 (m, 1H), 0.89–0.97 (m, 18H), 0.66–0.73 (m, 6H); ^13^C-NMR (125 MHz, CDCl_3_), δ (ppm): 175.9, 171.7, 171.5, 170.1, 170.0, 136.2, 129.1(2C), 128.8(2C), 127.2, 57.8, 53.7(2C), 48.7, 47.8, 41.5, 41.1, 37.7, 37.5, 34.5, 31.4, 29.5, 24.9(2C), 24.8, 24.5, 23.2, 23.1, 22.9(2C), 22.2, 21.9, 21.8, 21.6; MS (ESI) *m*/*z*: 628.8 [M + H]^+^, 645.9 [M + NH4]^+^, 650.8 [M + Na]^+^.

#### 4.1.18. Cyclo(Me-d-Leu-Leu-Leu-Me-d-Phe-Leu) (**A3**)

Yield: 44.5% ; white powder (CH_3_OH); ^1^H-NMR (500 MHz, CDCl3), δ (ppm): 8.17(d, *J* = 10.0 Hz, 1H), 7.07–7.23 (m, 5H), 6.85 (d, *J* = 5.0 Hz, 1H), 6.69 (d, *J* = 10.0 Hz, 1H), 5.23 (dd, *J* = 5.0 Hz, *J* = 5.0 Hz, 1H), 4.83 (dd, *J* = 5.0 Hz, *J* = 5.0 Hz, 1H), 4.68 (dd, *J* = 5.0 Hz, *J* = 5.0 Hz, 1H), 4.50 (dd, *J* = 10.0 Hz, *J* = 10.0 Hz, 1H), 3.47 (t, *J* = 20.0 Hz, 1H), 3.2 (s, 3H), 3.11 (s, 1H), 3.03 (d, *J* = 10.0 Hz, 2H), 2.97 (s, 3H), 2.12 (s, 1H), 1.74–1.84 (m, 4H), 1.49–1.52 (m, 2H), 1.32–1.39 (m, 4H), 0.76–1.00 (m, 24H); ^13^C-NMR (125 MHz, CDCl3), δ (ppm): 173.6, 173.2, 171.5, 170.1, 169.7, 136.3, 129.6(2C), 128.5(2C), 126.9, 57.5, 54.3, 52.6, 47.8, 46.8, 40.7(2C), 39.2, 37.5, 37.1, 31.3, 31.2, 29.6, 24.8, 24.4, 23.6, 23.3(2C), 23.0, 22.6(2C), 22.2, 22.0, 21.8; MS (ESI) *m*/*z*: 628.6 [M+H]^+^, 645.7 [M + NH4]^+^, 650.4 [M + Na]^+^.

#### 4.1.19. Cyclo(Me-d-Leu-Leu-d-Leu-Me-d-Phe-Leu) (**A4**)

Yield: 43.3%; white powder (CH_3_OH); ^1^H-NMR (500 MHz, CDCl_3_), δ (ppm): 7.20–7.27 (m, 4H), 7.06 (d, *J* = 10.0 Hz, 1H), 6.85 (d, *J* = 10.0 Hz, 1H), 6.40 (m, 1H), 6.17 (t, 1H), 5.45 (dd, *J* = 5.0, 5.0 Hz, 1H), 4.99 (td, 1H), 4.68 (td, 1H), 4.41–4.49 (m, 2H), 3.20–3.27 (m, 4H), 3.07 (d, *J* = 5.0 Hz, 1H), 3.01–3.07 (m, 1H), 2.96–2.99 (m, 1H), 2.78 (d, *J* = 5.0 Hz, 3H), 1.81 (dd, *J* = 5.0, 5.0 Hz, 2H), 1.59–1.62 (m, 4H), 1.43–1.48 (m, 2H), 1.30 (dd, *J* = 10.0, 10.0 Hz, 2H), 0.98–1.03 (m, 6H), 0.93 (dd, *J* = 10.0, 10.0 Hz, 12H), 0.84 (d, *J* = 5.0 Hz, 3H), 0.76 (d, *J* = 5.0 Hz, 3H); ^13^C-NMR (125 MHz, CDCl_3_), δ (ppm): 175.7, 172.3, 171.4(2C), 169.5, 136.8(3C), 128.8(2C), 126.8, 57.2, 56.2(2C), 52.6(3C), 48.4(2C), 48.0(2C), 41.3, 40.9, 37.5(3C), 33.9, 30.9(2C), 29.9(2C), 25.2, 23.1, 22.8, 22.7; MS (ESI) *m*/*z*: 628.7 [M + H]^+^, 645.8 [M + NH_4_]^+^, 650.6 [M + Na]^+^.

#### 4.1.20. Cyclo(Leu-d-*N*-Me-Phe-d-Leu-d-*N*-Me-Leu-d-Leu) (**A5**)

Yield: 47.0%; white powder (CH_3_OH); ^1^H-NMR (500 MHz, CDCl_3_), δ (ppm): 7.22–7.31 (m, 5H), 7.06 (d, *J* = 10.0 Hz, 1H), 6.95 (d, *J* = 10.0 Hz, 1H), 6.07 (d, *J* = 10.0 Hz, 1H), 5.17 (t, *J* = 10.0 Hz, 1H), 4.82 (m, 3H), 3.33 (m, 1H), 3.20 (m, 5H), 3.02 (d, *J* = 10.0 Hz, 1H), 2.66 (s, 3H), 1.95–2.00 (m, 3H), 1.77–1.85 (m, 2H), 1.66–1.75 (m, 2H), 1.47–1.52 (m, 3H), 1.2 (dd, *J* = 5.0, 5.0 Hz, 1H), 0.81–0.95 (m, 24H); ^13^C-NMR (125 MHz, CDCl_3_), δ (ppm): 173.4, 172.9, 172.1, 171.4, 168.5, 138.8, 129.5(2C), 128.8, 128.7, 127, 68.36(2C), 59.37, 55.37, 47.66, 47.3, 41.7, 36.0(3C), 30.8(2C), 25.4(3C), 23.0(3C), 22.6(3C), 22.03(2C); MS (ESI) *m*/*z*: 628.6[M + H]^+^, 645.7 [M + NH_4_]^+^, 650.6 [M + Na]^+^.

### 4.2. Antitumor Activity in Vitro

#### 4.2.1. Cell Culture

Human hepatocellular carcinoma HepG2, liver L02, colon carcinoma SW480, breast cancer MCF-7 and glioma U87 cells were obtained from the China cell bank of the Institute of Biochemistry and Cell Biology and maintained in Dulbecco’s modification Eagle’s medium (DMEM, Corning, NY, USA) with 10% fetal bovine serum (FBS, Hyclone, Logan, UT, USA), 1% penicillin-streptomycin and an antifungal agent. All of the cells were incubated at 37 °C in a humidified atmosphere composed of 95% air and 5% CO_2_.

#### 4.2.2. Anti-Proliferative Activity Using MTT Assays

The cell viability was determined by measuring the ability of cells to transform MTT (Genview, Houston, TX, USA) to a purple formazan dye. The cells were seeded in 96-well tissue culture plates at 2.5 × 10^3^ cells/well for 24 h. The cells were then incubated with galaxamide and its analogues at different concentrations for 72 h. After incubation, 20 μL/well of MTT solution (5 mg/mL phosphate buffered saline) were added and incubated for 5 h. The medium was aspirated and replaced with 100 μL/well of DMSO to dissolve the formazan salt. The color intensity of the formazan solution, which reflects the cell growth condition, was measured at 570 nm using a microplate spectrophotometer (SpectroAma™ 250, Winooski, VT, USA).

#### 4.2.3. Cell Apoptosis Analysis

The apoptotic cells were quantified using an Annexin V-FITC (Sigma-Aldrich, USA) Apoptosis Detection Kit according to the manufacturer’s instructions. Briefly, the cells were plated in a 6-well plates and treated with galaxamide and **A5** (6 μg/mL, 12 μg/mL, 24 μg/mL) for 72 h before being collected and resuspended in 200 μL of binding buffer. Five μL of Annexin V-fluorescein isothiocyanate (FITC) and 5 μL of PI were added. The analysis was performed with a FACScan flow cytometer (Coulter Epics Elite, Miami, FL, USA). The cells in the FITC-positive and PI-negative fraction were regarded as apoptotic cells.

#### 4.2.4. Observations of Nuclear Damage

Exponentially growing HepG2 cells (1 × 10^5^ cells/mL) were incubated for 72 h with 3, 6, 12 μg/mL of galaxamide and **A5**, respectively. Apoptotic nuclear morphology was visualized by the Hoechsst33342 staining technique. Cells were fixed with 3.7% of paraformaldehyde for 10 min at room temperature and washed three times with PBS. Thus, paraformaldehyde-fixed cells were stained using Hoechsst33342 (10 μg/mL) in the dark for 10 min. After washing three times with PBS, cells were visualized using fluorescence microscope.

#### 4.2.5. Western Blot Analysis

The protein was extracted with RIPA buffer and quantified using the Bradford method. Equal amounts of protein were loaded into each well. After SDS-PAGE, the proteins were transferred to a polyvinylidene difluoride (PVDF) membrane. The membrane was blocked with 5% non-fat milk for 1 h and incubated with a primary antibody (Cell Signaling Technology, Danvers, MA, USA, Rabbit monoclonal antibody, 1:1000) at 4 °C overnight. After washing two times with TBST (Tris-buffered saline with 0.1% Tween-20) buffer, the membrane was incubated with a secondary antibody (goat-anti-rabbit horseradish peroxidase (HRP)-conjugated 1:15,000) at room temperature for 1 h. The membranes were washed three times (8 min each time) with TBST. The intensity of the specific immunoreactive bands was detected through enhanced chemiluminescence (ECL).

#### 4.2.6. Statistical Analysis

The data are expressed as the mean ± standard error of mean (SEM). The statistical comparisons were performed using the Student’s *t*-test, and the differences between groups were considered significant at *p* < 0.05.

## 5. Conclusions

This paper has reported the synthesis and the biological evaluation of novel cyclopeptides, including galaxamide and its analogues **A1**–**A5**, against a series of cancer cells and normal liver cells. Some modified analogues exhibited strong cytotoxicity against cancer cells. Meanwhile, galaxamide and **A5** activated an apoptotic pathway involving the sequential activation of caspases 9, 3 and PARP in the HepG2 cells. Interest in exploring the chemical structure relationship of galaxamide and analogues with anti-tumor properties for new drug design has grown.
