# Correction: Xiao, X., *et al*. Synthesis, Cytotoxicity and Apoptosis Induction in Human Tumor Cells by Galaxamide and Its Analogues. *Mar. Drugs* 2014, *12*, 4521–4538

**DOI:** 10.3390/md12105123

**Published:** 2014-09-30

**Authors:** Xi Xiao, Xiaojian Liao, Shaoling Qiu, Zihao Liu, Bin Du, Shihai Xu

**Affiliations:** 1 Department of Chemistry, Life Science School, Jinan University, Guangzhou 510632, China; E-Mails: xxiaoxi0312@163.com (X.X.); tliaoxj@jnu.edu.cn (X.L.); qslhappyrain@gmail.com (S.Q.); 2 Department of Pathology, Medical School, Jinan University, Guangzhou 510632, China; E-Mail: mercuryliu@sina.cn

We have found the following error in the title of this article, which was recently published in *Mar. Drugs* [1]: a word “paper” has been inserted at the beginning of title by mistake. The correct title should be: Synthesis, Cytotoxicity and Apoptosis Induction in Human Tumor Cells by Galaxamide and Its Analogues.

We apologize for any inconvenience caused to the readers.
